# Supplementary material for: Attitude to Co-Administration of Influenza and COVID-19 Vaccines among Pregnant Women Exploring the Health Action Process Approach Model
Source: Vaccines (Basel). 2024 Apr 28;12(5):470. doi: 10.3390/vaccines12050470 (PMC11125974; doi:10.3390/vaccines12050470)
Supplement: Supplementary file 1 [file vaccines-12-00470-s001.zip › vaccines-2952206-supplementary.pdf]

Supplementary Materials

Attitude to Co-Administration of Influenza and COVID-19 Vaccines among Pregnant Women Exploring the Health Action Process Approach Model

Alessandra Fallucca [1,*], Palmira Immordino [1], Patrizia Ferro [1], Luca Mazzeo [1], Sefora Petta [1], Antonio Maiorana [2], Marianna Maranto [2], Alessandra Casuccio [1] and Vincenzo Restivo [3]

[1] Department of Health Promotion, Mother and Child Care, Internal Medicine and Medical Specialities, University of Palermo, 90127 Palermo, Italy; palmira.immordino@unipa.it (P.I.); patrizia.ferro@unipa.it (P.F.); luca.mazzeo@unipa.it (L.M.); sefora.petta@gmail.com (S.P.); alessandra.casuccio@unipa.it (A.C.)

[2] HCU Obstetrics and Gynaecology, ARNAS Civico Di Cristina—Benfratelli Hospital, 90127 Palermo, Italy; antonio.maiorana@arnascivico.it (A.M.); marianna.maranto@arnascivico.it (M.M.)

[3] School of Medicine, University Kore of Enna, 94100 Enna, Italy; vincenzo.restivo@unikore.it

* Correspondence: alessandra.fallucca@unipa.it; Tel.: +39-0916553630 or +39-3804703272

Contents

S1: English version of the questionnaire page 2

S2: Italian version of the questionnaire page 4

Università degli Studi di Palermo

Civico Di Cristina Benfratelli
Azienda di Rilievo Nazionale ad Alta Specializzazione

Survey

Attitude of pregnant women to receive co-administration of influenza and COVID-19

SECTION A

1) How old are you??

____

2) What is your nationality?
☐ Italian
☐ Foreign

3) Where are you resident??
☐ Palermo city
☐ Province of Palermo
☐ Other Sicilian cities
☐ Other Italian cities

4) What is your marital status??
☐ Single
☐ Engaged
☐ Married
☐ Divorced
☐ Cohabitant

5) What is your educational qualification??
☐ Primary school diploma
☐ Secondary school diploma
☐ High school diploma
☐ Degree/Higher qualification

6) What is your occupation?
☐ Employed
☐ Unemployed
☐ Housewife

7) What week of pregnancy are you??________

8) How many childrens do you have?_________

9) Is your pregnancy at risk??
☐ Yes
☐ No

10) If your answer is yes, what is the reason for the risk?

____

11) What was your main source of information about vaccines?
☐ Gynecologist
☐ Family clinic health worker
☐ General practitioner
☐ Pediatrician
☐ Vaccination centre health worker
☐ Friends
☐ Family
☐ TV/Media
☐ Web/Internet
☐Other

____

12) Would you receive the influenza and the COVID-19 vaccine coadministered during pregnancy??
☐ Yes
☐ No

SECTION B – Health Action Process Approach model

Risk perception	Strongly agree	Agree	Undecided	Disagree	Strongly disagree
SARS-CoV-2 and influenza infection could increase the risk of miscarriage					
SARS-CoV-2 and influenza infection could increase the risk of resorting to cesarean delivery					

Positive outcomes	Strongly agree	Agree	Undecided	Disagree	Strongly disagree
Co-administration of COVID-19 and infuenza vaccines could reduce the risk of being hospitalized for complications					
Co-administration of COVID-19 and infuenza vaccines could protect my baby in few first months of life					

Negative outcomes	Strongly agree	Agree	Undecided	Disagree	Strongly disagree
Co-administration of COVID-19 and infuenza vaccines could lead to the same frequency of side effects , such as fever or headache, as if the individual vaccines were given separately					
Co-administration of COVID-19 and infuenza vaccines could lead to the same frequency of side effects , such as pain, redness and swelling in the arms despite of given the individual vaccines					

Self efficacy	Strongly agree	Agree	Undecided	Disagree	Strongly disagree
I am confident to have enough informations about co-administration of COVID19 and influenza vaccines to make the decision to get them					
I am sure to have co-administration even though my family/friends disagree					

Università degli Studi di Palermo

Civico Di Cristina Benfratelli
Azienda di Rilievo Nazionale ad Alta Specializzazione

Questionario

Attitudine delle donne in gravidanza nei confronti della vaccinazione anti-influenzale e anti-SARS-COV2

SEZIONE A

1) Quanti anni ha?

____

2) Qual è la sua nazionalità?
   - ▢ Italiana
   - ▢ Estera

3) Dove è residente?
   - ▢ Palermo
   - ▢ Provincia di Palermo
   - ▢ Altre città siciliane
   - ▢ Altre città italiane

4) Qual è il suo stato civile attuale?
   - ▢ Nubile
   - ▢ Fidanzata
   - ▢ Sposata
   - ▢ Separata/Divorziata
   - ▢ Convivente

5) Qual è il suo titolo di studio?
   - ▢ Licenza elementare
   - ▢ Licenza media
   - ▢ Diploma di scuola superiore
   - ▢ Laurea/ Titolo superiore

6) Che attività lavorativa svolge?
   - ▢ Dipendente/impiegata
   - ▢ Disoccupata
   - ▢ Casalinga

7) In che settimana di gravidanza si trova?____

8) Quanti figli ha?__________________________

9) La sua è una gravidanza a rischio?
   - ▢ Sì
   - ▢ No

10) Se sì, per quale ragione?

____

11) Quale è stata la sua principale fonte di informazione sulle vaccinazioni?
   - ▢ Ginecologo
   - ▢ Operatore sanitario del consultorio
   - ▢ Medico di Medicina Generale
   - ▢ Pediatra
   - ▢ Operatore sanitario del centro vaccinale
   - ▢ Amici
   - ▢ Parenti
   - ▢ TV/ Media
   - ▢ Web/ Internet
   - ▢ Altro

____

12) Durante la gravidanza riceverebbe una somministrazione contemporanea di due vaccini?
   - ▢ Sì
   - ▢ No

SEZIONE B – Health Action Process Approach model

Percezione del rischio	Molto d’accordo	D’accordo	Indeciso	Disaccordo	Per niente d’accordo
L’infezione da SARS-CoV-2 e virus influenzale potrebbe aumentare il rischio di aborto					
L’infezione da SARS-CoV-2 e virus influenzale potrebbe aumentare il rischio di ricorso a parto cesareo					

Aspettative di esito positivo	Molto d’accordo	D’accordo	Indeciso	Disaccordo	Per niente d’accordo
La co-somministrazione dei vaccini contro COVID-19 e influenza potrebbe ridurre il rischio di essere ricoverata per complicanze legate alle malattie					
La co-somministrazione dei vaccini contro il COVID-19 e l’influenza potrebbe proteggere il mio bambino dalle infezioni nei primi mesi di vita					

Aspettative di esito negativo	Molto d’accordo	D’accordo	Indeciso	Disaccordo	Per niente d’accordo
La co-somministrazione dei vaccini contro il COVID-19 e l’influenza può determinare la stessa frequenza di effetti indesiderati come febbre o mal di testa rispetto alla somministrazione separata dei singoli vaccini					
La co-somministrazione dei vaccini contro il COVID-19 e l’influenza può determinare la stessa frequenza di effetti indesiderati come dolore, rossore e gonfiore alle braccia rispetto alla somministrazione separata dei singoli vaccini					

Autoefficacia	Molto d’accordo	D’accordo	Indeciso	Disaccordo	Per niente d’accordo
Sono sicura di avere abbastanza informazioni sulla co-somministrazione dei vaccini contro COVID-19 e influenza per poter decidere di farli					
Sono sicura di potermi sottoporre alla co-somministrazione anche qualora i miei familiari/amici non siano d’accordo:					
